# Attention Combines Similarly in Covert and Overt Conditions

**DOI:** 10.3390/vision3020016

**Published:** 2019-04-25

**Authors:** Christopher D. Blair, Jelena Ristic

**Affiliations:** 1 Department of Psychology, Eastern Oregon University, La Grande, OR 97850, USA; 2 Department of Psychology, McGill University, Montreal, QC H3A 1B1, Canada

**Keywords:** attention, covert, overt, combined, automated, voluntary

## Abstract

Attention is classically classified according to mode of engagement into voluntary and reflexive, and type of operation into covert and overt. The first distinguishes whether attention is elicited intentionally or by unexpected events; the second, whether attention is directed with or without eye movements. Recently, this taxonomy has been expanded to include automated orienting engaged by overlearned symbols and combined attention engaged by a combination of several modes of function. However, so far, combined effects were demonstrated in covert conditions only, and, thus, here we examined if attentional modes combined in overt responses as well. To do so, we elicited automated, voluntary, and combined orienting in covert, i.e., when participants responded manually and maintained central fixation, and overt cases, i.e., when they responded by looking. The data indicated typical effects for automated and voluntary conditions in both covert and overt data, with the magnitudes of the combined effect larger than the magnitude of each mode alone as well as their additive sum. No differences in the combined effects emerged across covert and overt conditions. As such, these results show that attentional systems combine similarly in covert and overt responses and highlight attention’s dynamic flexibility in facilitating human behavior.

## 1. Introduction

Attention is often classified as operating in two modes—*reflexive* and *voluntary*—and two types —*covert* and *overt* [1]. The first distinction reflects the way in which attention is engaged, either by unpredictable events in the environment in a reflexive manner [2,3] or by internal goals of an individual in a voluntary manner (e.g., Reference [4]). Reflexive attention is typically engaged quickly by 100–300 ms but it also subsides quickly by 500 ms. Voluntary attention, in contrast, takes longer to emerge, about 300–500 ms, but it lasts longer for about 1000 ms (e.g., References [5,6]). The second distinction reflects the way in which either of these two modes may be reflected, covertly, while participants respond to target items with manual key presses and keep their eyes fixated on a central location [1,7,8,9] or overtly, while they saccade to targets and withhold manual responses (e.g., References [10,11]). Covert attention is thought to reflect the alignment of mental attentional resources with the response target while overt attention additionally allows for an alignment of oculomotor resources with that response target.

Recently, this classic taxonomy has been expanded to accommodate new attentional modes, namely those that show control of attention by social cues like eye gaze (e.g., References [12,13,14,15]), reward (e.g., Reference [16]), and overlearned symbols that carry a history of selection, such as arrows (e.g., References [17,18,19,20,21,22,23]). Ristic and Kingstone [22,23] were among the first to show that attention directed by a task-irrelevant arrow operated independently and in parallel with the known reflexive and voluntary modes. Using a double simultaneous cuing task in which this automated cue was paired either with a typical reflexive, i.e., an abrupt peripheral onset, or a typical voluntary attention cue, i.e., a shape symbol, the authors found no costs to either form of orienting and additive effects when both cues indicated the same target location. Based on these and other subsequent findings (e.g., References [24,25,26]), it has been argued that automated orienting facilitates the processing of behaviorally relevant stimuli in the environment in order to aid complex behaviors. 

In support of this notion, when symbolic cues like arrows are made task-relevant, such that they carry predictive value about the response target, attentional resources available to both automated and voluntary systems are found to combine (e.g., References [21,27,28,29,30]). Ristic and Landry [21] demonstrated this result (see also Reference [30]) by eliciting automated attention using spatially non-predictive arrow cues and voluntary attention using spatially informative non-directional shapes. Both cues produced typical effects in that non-predictive arrows engaged attention quickly and persistently while central shapes engaged voluntary attention slowly in an effortful manner. However, when arrow cues were made spatially predictive, and thus relevant for the task at hand, the magnitude of the observed orienting effect exceeded the isolated magnitudes of each automated and voluntary orienting, as well as their additive combination. That is, automated and voluntary systems combined superadditively when the cue was both behaviorally relevant, as it was an overlearned symbol, and task relevant, as it was spatially informative about the target. Using EEG, Blair and Ristic [27] recently reported that these superadditive effects appear not to reflect underlying combined increases in the early cortical processing of the target, but rather the suppression of irrelevant responses, as indicated by the increases in the synchrony of target-related activity in the alpha frequency and diminished modulation of the P1 sensory component.

While, together, these data show the dynamic flexibility of attentional systems when attention is elicited covertly, it remains unknown whether similar findings would be observed if participants were required make overt instead of covert responses. Covert and overt attention have typically been found to operate in a similar, but dissociable, manner, such that, like manual response time, saccadic reaction time (SRT), or the time it takes the participant to look at the target, is shorter for targets cued by the attentional cue relative to those not cued [31,32]. However, although covert and overt attention rely on different response systems (manual vs. oculomotor), they nevertheless engage partially overlapping neural networks. This has been demonstrated in studies examining the neural correlates of tasks involving the movements of covert visual attention and overt movements of the eyes and attention together (e.g., References [33,34,35]), with the overlap in activity observed in the frontal eye field (FEF) and supplementary eye field (SEF), as well as a collection of parietal and temporal regions [35]. However, while fMRI data have shown upwards of 60%–80% overlap in areas responsible for both types of attention (e.g., Reference [35]), some areas appear to be uniquely activated when a saccade is executed, such as the posterior vermis of the cerebellum while other areas show a greater degree of activation for one or the other type, such as the frontal cortex for covert shifts of attention and areas of the occipital cortex for overt shifts of attention [11,33,35,36]. As such, these findings suggest a functional relationship between covert and overt attention, but do not rule out the possibility that the same areas may be recruited in different ways, and/or that different neurons within the same general areas may be differentially activated in each type of orienting. 

In the present study, we assessed if attention combined similarly in covert and overt conditions. To do so, we employed the same experimental task as Ristic and Landry [21] while monitoring and measuring participants’ eye movements using a high-speed remote eye tracker. Based on past data showing similarities between covert and overt attentional systems, we hypothesized that automated attention would combine with voluntary attention in a similar manner across both response conditions, consistent with the idea that covert and overt attentional systems operate in a flexible, rather than restricted, manner, as while there may be some separation in the functioning of these two systems, it appears this does not require them to operate by completely different mechanisms.

## 2. Materials and Methods 

### 2.1. Participants

Nineteen adult volunteers (14 female, mean age = 22.11; SD = 3.81) participated. All were naïve as to the purpose of the experiment, reported normal or corrected-to normal vision, and no history of neurological or psychiatric disorders. Participants were students at McGill University and participated in exchange for course credit. All procedures were approved by the University’s Research Ethics Board. Informed consent was obtained from all participants (project identification code #81-0909). We used G_Power 3.1 [37] to estimate the average sample size needed to detect an effect of cue validity on participant response times in automated, voluntary, and combined attention conditions. We estimated this effect size from our previous work using a similar paradigm to assess attentional orienting effects in automated, voluntary, and combined circumstances [27]. Cohen’s standardized difference scores (dz), reflecting contrasts between validly and invalidly cued conditions were estimated using paired-sample t test values and sample sizes derived from the previous dataset (i.e., d_z_ = t/√N [38,39]). All power calculations reflected two-tailed probabilities and α of 0.05. The average effect size found in the previous study across all three attention conditions was small to medium in magnitude at a value of 0.37. As such, samples sizes of 17 participants would be required to yield 0.80 power, whereas a sample of 25 would be required to yield 0.95 power.

### 2.2. Apparatus and Stimuli

**Figure 1:**
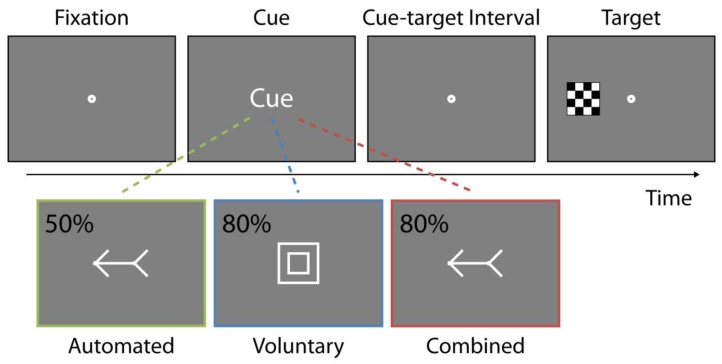
Stimuli and an example trial sequence. Each trial began with a fixation screen for 600 ms. Then, depending on the condition, a central shape or arrow cue, indicating a left or right target location, was presented for 100 ms. The display then reverted to the fixation screen for a randomly determined time interval ranging from 250 to 650 ms, thus making up the 350 to 750 ms cue-target time intervals. A response target was presented peripherally on the left or right of fixation and remained on the screen until response was made or 2700 ms had elapsed. Note that stimuli are not drawn to scale and that condition color-coding was not used during the procedure. Note that percentages listed for each attention condition indicate the accuracy of the cue in indicating the correct location of the upcoming target.

As illustrated in Figure 1, stimuli were white line drawings and included an arrow, geometric shapes, fixation point, and a black and a white checkerboard target. The stimuli were presented against a 50% gray background on a 16 inch Cathode Ray Tube (CRT) monitor at an approximate viewing distance of 63 cm. Arrow cues (3.3° in length) were comprised of a horizontal straight line (2.4°) with an arrowhead and an arrowtail (each 0.9° in length). Shape cues were a square and a circle with an outer frame measuring 2.4° and the inner frame measuring 1.92° while the fixation point was comprised of an outer white circle (0.68°) and inner gray circle (0.17°). The response target was a 0.96° square made up of black and white squares (each measuring 0.24°). The target appeared 7.5° to the left or right of fixation along the horizontal axis. All stimuli were created and presented using the Psychophysics Toolbox [40,41] for MATLAB (Mathworks Inc., Natick, MA).

Eye movements were recorded using a remote SR Research EyeLink 1000 eye tracker, sampling with a temporal resolution of 500Hz and a spatial resolution of at least 1°. Although viewing was binocular, only the right eye was recorded. Nine-point calibration and drift correction were performed before each testing session and repeated throughout as needed (e.g., Reference [42]).

### 2.3. Design

The study was a fully repeated measures design with response modality (manual, oculomotor; blocked and counterbalanced), attention condition (automated, voluntary, combined; blocked and counterbalanced), cue validity (valid, invalid; intermixed), and cue-target time interval (short, long; intermixed) included as variables. A fourth condition, in which shape cues were presented as spatially uninformative about the target’s location, was included as a control to ensure that these cues only engaged attention when manipulated as spatially predictive of target location. The data confirmed this notion. A repeated-measures ANOVA with response modality, cue validity, and cue-target interval included as factors indicated no significant effect of cue validity (main effect F(1,18) = 0.10, *p* = 0.76), and no interactions between cue validity and any other factor (all Fs < 0.62, ps > 0.44).

Response modality reflected the type of attention with which participants were instructed to respond. In the manual condition, participants were instructed to press the spacebar as quickly as possible when a target appeared and to withhold eye movements. In the oculomotor condition, participants were instructed to look at the target as quickly as possible when it appeared and to withhold manual responses. Response modality was blocked across the two testing sessions, which occurred at least one day apart (average of five days, with 63% of participants completing the study within two days), and counterbalanced for order of presentation such that half of participants received the oculomotor condition on day one, while the other half received the manual condition on day one.

Within each session, participants completed all attention conditions, which were also counterbalanced for presentation order. Attention condition reflected the type of attentional control elicited by the cues with the tasks reflecting the standard procedures and parameters in the literature (e.g., References [20,21,27,43,44,45]). Automated attention was engaged using central spatially non-predictive arrows. Arrow cues pointed to the left or right and indicated the correct side of target appearance equally often (*p* = 0.5). Voluntary attention was engaged using central spatially predictive shapes. Shape cues predicted the correct target location with a probability of 80% (*p* = 0.8). For each participant, the square or circle stimulus was designated as indicating that the target was more likely to appear on the left side of the screen, or vice versa. Combined attention was engaged using central spatially predictive arrows, which indicated the correct target location with 80% probability (*p* = 0.8; see Figure 1). At the beginning of each attention condition, participants were instructed about the response modality and were informed, and it was verified that they understood, about the predictive probability associated with the cues.

The combination of cue’s direction and target’s position made up the cue validity factor, with valid trials those in which the cue indicated the same spatial location as the location of the subsequent target, and invalid trials those in which the cue indicated a different spatial location than the subsequent target.

A range of cue-target time intervals was used in order capture the temporal profiles of attentional effects. Automated orienting has been shown to emerge relatively quickly, within 100–300 ms and to last about 700–1000 ms [22,23], while voluntary orienting may take up to 500 ms to emerge (e.g., References [5,6]). Combined orienting typically emerges as early as automated orienting [21] and also persists for up to 1 s, due to voluntary attention influences. We manipulated cue-target delay times between 350 and 750 ms, with the specific time interval for each trial drawn from a rectangular distribution of all values. For analysis purposes, the time delays between 350 and 550 ms were classified as short, while those between 550 and 750 ms were classified as long. Within each condition, all cue-target time intervals, cue direction, and target location combinations were presented equally often and in a pseudorandom sequence that varied from participant to participant.

### 2.4. Procedure

As illustrated in Figure 1, each trial began with a 600 ms fixation display, after which an attentional cue, either an arrow (left or right) or geometric shape (circle or square), depending on the condition, was presented for 100 ms. The screen then reverted to the fixation display for the remaining 250–650 ms of the cue-target time interval. The response target was presented to the left or right of fixation and remained visible until participants responded or 2700 ms had elapsed, whichever came first. About 5% of trials contained no target and participants were instructed to withhold all responses.

Responses were measured from target onset. For oculomotor sessions, saccadic response time (SRT) was recorded when the saccade reached a point within 2° of the target. If the eye tracker detected that participants had broken fixation at any point during the trial in both response conditions, other than at the point when an eye movement response was required (i.e., the target was presented in the oculomotor condition), the trial was aborted and restarted after the message “*Please Maintain Fixation*” or “*Please Maintain Fixation, except when the target appears*” was displayed, depending on the response modality. Fixation was considered broken if a participant’s gaze was over 2° horizontally and/or 3° vertically away from fixation. Saccades were defined as eye movements with a minimum velocity of 30°/sec.

At the start of each session, participants were comfortably positioned in a chin rest, received instructions, and after the calibration procedure, which was repeated before each attention condition and whenever participants removed their head from the chin rest for any reason throughout the course of the study, completed 10 practice trials. There were 210 trials for each attention condition and response modality combination, for a total of 1260 trials.

## 3. Results

### 3.1. Data Handling

Trials on which participants responded in less than 100 ms were marked as anticipations (manual: Automated 2.76%, voluntary 2.61%, combined 2.11%; oculomotor: Automated 0.18%, voluntary 0.40%, combined 0.50%). Trials on which participants took longer to respond than two standard deviations above their own mean response time were marked as timed-out responses (manual: Automated 3.11%, voluntary 3.24%, combined 2.90%; oculomotor: Automated 3.87%, voluntary 3.53%, combined 3.47%). For this purpose, separate average response times and variability were calculated for each participant as a function of response type and attention condition. No-target trials on which participants made a manual or oculomotor response were marked as false alarms (manual: Automated 8.95%, voluntary 9.47%, combined 14.21%; oculomotor: Automated 24.74%, voluntary 17.90%, combined 24.21%). We note the relatively high rate of false alarms, which may be due in part to the relatively high task demands making it difficult for participants to suppress responses when no target was present. However, importantly, there were no significant differences in false alarm rates between attention conditions (automated, voluntary, combined, all two-tailed pairwise t-tests, ts < 1.7, ps >.1), suggesting that task demands were equated. Error rates did not differ across conditions (all two-tailed pairwise t-tests, ts < 2, ps > 0.07). The percentage of trials containing any errors (anticipations, timeouts, false alarms) was as follows for each condition: Manual: Automated 6.02%, Voluntary 6.02%, Combined 5.44%; oculomotor: Automated 5.04%, Voluntary 4.59%, Combined 4.94%. To ensure that false alarm rates did not vary as a function of previous trial validity, we used paired two-tailed t-tests to compare the proportion of false alarms that occurred on no-target trials after a valid trial with the proportion of false alarms that occurred on no-target trials after an invalid trial separately for each response condition and attention type as well as using an overall comparison. No differences were found (manual condition, all ts < 0.4, ps > 0.12; oculomotor condition, all ts < 1.9, ps > 0.07; overall comparison *t*(18) = 1.23, *p* = 0.235). All trials containing anticipations or timed-out responses were excluded from further analyses.

Three types of analyses were performed. First, we verified that different modes of attention were engaged in each condition by examining mean correct interparticipant response times in an omnibus repeated measures ANOVA with factors of response type (manual, oculomotor), attention condition (automated, voluntary, combined), cue validity (validly cued, invalidly cued), and cue target time interval (short, long). Second, to establish that each attention condition produced expected results, mean correct interparticipant response times were also examined using separate ANOVAs for each attention condition as a function of response type, cue validity, and cue target time interval. Finally, we analyzed effect magnitudes across attention conditions assessing if attention combined similarly across the two response types. Paired sample t-tests were conducted to follow up interactions when appropriate, with the p-values corrected for multiple comparisons using a Bonferroni procedure.

### 3.2. Omnibus Analyses

**Figure 2:**
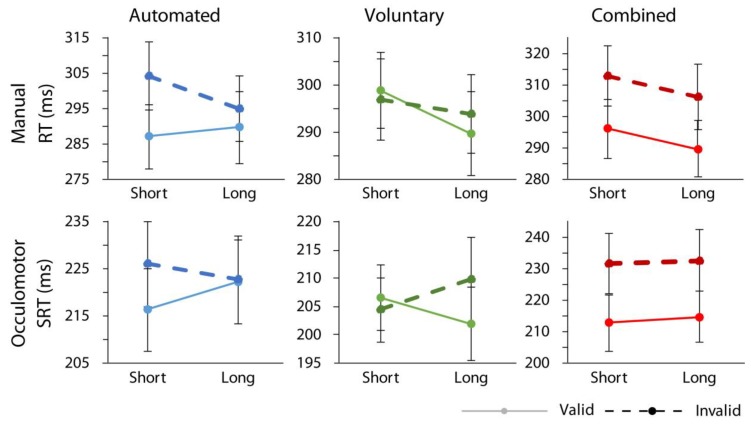
Response time data. Mean correct interparticipant response times plotted as a function of attention condition, response modality, cue-target time interval, and cue validity. Error bars indicate standard error of the difference between means.

The ANOVA returned a significant main effect of cue validity, F(1,18) = 29.07, *p* < 0.001, ηp2 = 0.62, showing that overall, participants responded faster on validly cued relative to invalidly cued trials. Furthermore, there was a significant interaction between attention condition and cue validity, F(2,36) = 33.29, *p* < 0.001, ηp2 = 0.65, confirming that the greatest RT difference between validly cued and invalidly cued trials was found in the combined case. Importantly, no significant interactions involving cue validity and response modality were found confirming that similar data patterns held for both manual and oculomotor responses as illustrated in Figure 2 (response modality x cue validity, F < 1; response modality x attention condition x cue validity, F(2,36) = 1.33, *p* = 0.28; response modality x cue-target time x cue validity, F < 1; response modality x cue validity x attention condition x cue-target time, F < 1). The same pattern of data was found when data from the control condition, in which shape cues were manipulated as spatially nonpredictive, were included in the omnibus ANOVA. Specifically, there was still a significant main effect of cue validity, F(1,18) = 25.79, *p* < 0.001, ηp2 = 0.59, a significant interaction between attention condition and cue validity, F(3,54) = 28.82, *p* < 0.001, ηp2 = 0.62, and no significant interactions between response modality and cue validity, F(1,18) = 21.71, p = 0.698, or response modality, attention conditions, and cue validity, F(3,54) = 1.03, *p* = 0.388.

Though not of specific theoretical interest to our hypotheses, there were significant main effects of response modality, F(1,18) = 53.41, *p* < 0.001, ηp2 = 0.75, with participants responding faster overall in the oculomotor relative to manual condition, and attention condition, F(2,36) = 3.74, *p* = 0.034, ηp2 = 0.17, with participants responding, on average, the fastest in the voluntary attention condition, and slowest in the combined attention condition. There was also a significant interaction between response modality and cue-target time, F(1,18) = 6.71, *p* = 0.019, ηp2 = 0.27, with the steeper foreperiod effect in the manual relative to the oculomotor condition. There was an interaction between response modality and attention condition, F(2,36) = 3.55, *p* = 0.039, ηp2 = 0.17, whereby there were, on average, faster responses in the voluntary condition relative to the other two in the oculomotor response condition, but not the manual condition. An interaction between cue-target time, attention condition, and cue validity, F(2,36) = 14.40, *p* < 0.001, ηp2 = 0.44, was reliable as well, reflecting the larger orienting effects in the automated condition at short cue target times and larger orienting effects in the voluntary condition at long cue target times.

### 3.3. Attention Condition Analyses

#### 3.3.1. Automated Attention

As illustrated in Figure 2, and replicating past reports, automated attention produced expected results (e.g., References [21,22,23,27]). In particular, there were main effects of cue validity, F(1,18) = 19.86, *p* < 0.001, ηp2 = 0.52, with faster responses on validly cued relative to invalidly cued trials, and response modality, F(1,18) = 31.20, *p* < 0.001, ηp2 = 0.63, with faster overall responses in the oculomotor as compared to the manual condition. There was also a significant interaction between cue-target time interval and cue validity, F(1,18) = 23.94, *p* < 0.001, ηp2 = 0.57, indicating that the orienting effect was reliable at the short, *t*(18) = 6.96, *p* < 0.001, d = 0.49, but not long, *t*(18) = 1.31, *p* = 0.207, cue target time (with a Bonferroni corrected critical *p* of 0.025). No other effects or interactions were reliable (all Fs < 3.28, ps > 0.08).

#### 3.3.2. Voluntary Attention

The ANOVA examining the voluntary attention condition found no main effect of cue validity, F(1,18) = 1.50, *p* = 0.24, but indicated a reliable interaction between cue-target time interval and cue validity, F(1,18) = 11.76, *p* = 0.003, ηp2 = 0.40, demonstrating a significant orienting effect at the longer cue-target interval (long: *t*(18) = 3.88, *p* = 0.001, *d* = 0.30; short: *t*(18) = 0.99, *p* = 0.335). When voluntary orienting was examined for each response condition individually, the main effect of cue validity was not reliable in the manual condition, *t*(18) = 1.92, *p* = 0.071, but was significant in the oculomotor condition, t(18) = 2.49, *p* = 0.023, *d* = 0.26. A significant main effect of response modality, F(1,18) = 65.56, *p* < 0.001, ηp2 = 0.79, once again indicated overall faster responses in the oculomotor case (all other Fs < 3.6, ps > 0.07).

#### 3.3.3. Combined Attention

Finally, the combined condition returned significant main effects of cue validity, F(1,18) = 47.81, *p* < 0.001, ηp2 = 0.73, with overall faster responses on validly relative to invalidly cued trials [21,27,29] and response modality, F(1,18) = 54.72, *p* < 0.001, ηp2 = 0.75, with overall faster responses in the oculomotor condition. No other effects were reliable (all Fs < 3.22, ps > 0.08). Thus, each attention condition produced expected results in both manual and oculomotor conditions.

### 3.4. Additivity Analyses

**Figure 3:**
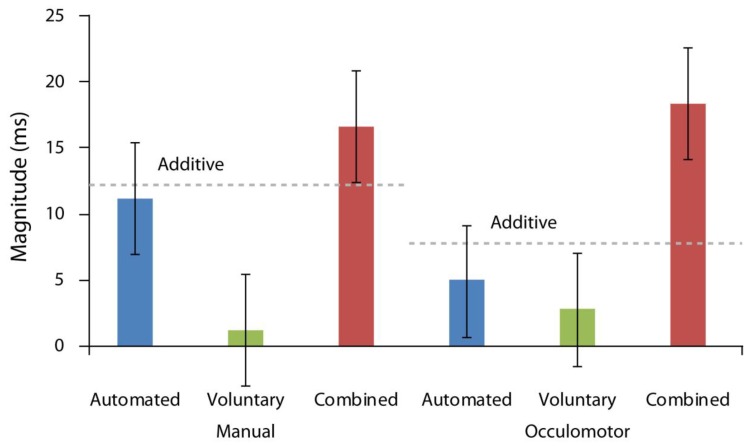
Additivity analyses. Magnitudes of attentional orienting (invalidly cued–validly cued response time) as a function of attention condition for manual and oculomotor conditions. Error bars indicate standard error of the difference between means.

First, we compared the magnitudes of orienting across all attention conditions. To do so, we calculated the magnitudes of orienting in each attention, response, and cue-target interval condition by subtracting valid from invalid response times. Then, we subjected these values to a repeated measures ANOVA run as a function of response modality, cue-target time interval, and attention condition included as factors. As illustrated in Figure 3, there was a significant main effect of attention condition, F(2,36) = 33.12, *p* < 0.001, ηp2 = 0.65, with the greatest orienting magnitudes found in the combined attention case. There was also a significant interaction between cue-target time interval and attention condition, F(3,36) = 14.44, *p* < 0.001, ηp2 = 0.45, reflecting greater orienting magnitudes for the automated condition on trials with shorter cue target intervals and for the voluntary condition on trials with longer cue target intervals. No other effects were reliable (all Fs < 1.33, ps > 0.27).

Finally, we examined whether automated and voluntary attention combined similarly in manual and oculomotor conditions. This analysis assessed whether the magnitude of attentional orienting in the combined condition was larger than the theoretically predicted additive sum of orienting magnitudes produced in the automated and voluntary attention conditions. To reiterate, one of the hallmarks of combined attention or attention elicited by a cue that carries both task relevant and behaviorally relevant meaning is that the magnitude of the observed orienting effect (invalid RT–valid RT) is greater than each individual effect’s magnitude in isolation and their additive sum, confirming an interactive relationship. To analyze for this, we performed an additional repeated measures ANOVA which compared the additive sum of automated and voluntary condition magnitudes with the measured combined attention effect as a function of response modality and cue-target time. The data, once again, indicated a significant main effect of attention condition, F(1,18) = 13.86, *p* = 0.002, ηp2 = 0.43, with the orienting magnitude in the combined case (M = 17.46 ms, SD = 10.28 ms) significantly larger than the orienting magnitude of the additive sum of automated and voluntary conditions (M = 10.24 ms, SD = 13.06 ms; [21,27,29]). Importantly, there was no interaction between response modality and attention condition, F(1,18) = 0.78, *p* = 0.39, or response modality, cue-target time, and attention condition, F(1,18) = 0.33, *p* = 0.57, indicating that these effects held equally for both manual and oculomotor responses (Figure 3).

To further inspect the plausibility that there was no interaction between response modality and attention condition, we also calculated the Bayes factor B [46,47,48,49,50], which indicates the relative strength of evidence for preferring the alternative hypothesis over the null. Values less than 0.33 indicate substantial evidence against the null, values between 0.33 and 3.0 indicate the need for more evidence, and values exceeding 3.0 indicate substantial evidence for the alternative hypothesis [48,51,52]. We evaluated the evidence for the interaction model against the main effects model. A JZS Bayes factor ANOVA [53] with default prior scales revealed that the main effects model (B = 0.92) was preferred to the interaction model (B = 0.4) by a Bayes factor of 2.3 (0.92/0.4). As such, this analysis provides supporting evidence for the null hypothesis and against the notion that response modality and attention condition interact.

## 4. Discussion

The aim of the present study was to investigate whether attention combined in a flexible fashion in covert and overt responses. Past work has shown that voluntary and reflexive attentional systems interact when behaviorally relevant cues, like arrows, are made task relevant (e.g., References [21,27,28,29,30]). However, these results were based on investigations of covert attention, when participants are required to maintain central fixation. Thus, it remained unknown whether similar effects would also be observed in overt conditions, when participants’ eye movements are measured. To address this question, here we elicited, measured, and compared automated, voluntary, and combined attention in both covert and overt conditions. We found expected effects for each attention case, with early effects in the automated case, slowly emerging effects in the voluntary case, and large superadditive effects in the combined case. Importantly, these effects occurred similarly in covert and overt conditions, with an overall tendency for participants to respond more quickly in the overt condition (also seen in past literature, e.g., Reference [10]), which was the only observed difference across overt and covert data. Note that while the potential delay between the keyboard key press and its registration by the computer was not quantified, any effects it may have had would be held constant across conditions and thus would be an unlikely contributing factor to the overall slower response times in the manual response condition as slower manual relative to oculomotor response times have been previously demonstrated in the literature [31].Thus, attention appears to combine similarly in covert and overt responses, suggesting that both types of attention operate in a flexible, rather than restricted, manner.

These results dovetail with the existing body of evidence that has shown that central symbolic cues, like arrows, facilitate both covert and overt attention in a voluntary saccade task (e.g., References [54,55,56,57,58]) and extend them to further demonstrate these effects in oculomotor conditions when arrows are used as attentional cues rather than as distractors. Specifically, past studies that have investigated overt automated orienting, have examined how directional cues like arrows [54,55,56,57,58] bias overt voluntary eye movements by manipulating arrow cues as distractors in cuing paradigms. While the majority of these studies show that such distractor cues lead to shorter saccadic latencies for cued trials and an increased number of directional saccade errors elicited in response to the cued side (with the exception of Reference [58]), they have nevertheless demonstrated this finding by manipulating arrows as distractors while participants’ task was to make voluntary saccades to a pre-specified target location. Here, we used the central arrows as attentional cues in an equivalent manner across covert and overt tasks, such that the cues in both conditions indicated target locations similarly and participants were instructed to respond to the target in an equivalent manner, i.e., by pressing a key or by looking. To our knowledge, this is the first demonstration of the effects of directional arrow cues in overt attention when arrows are manipulated as spatially nonpredictive and tested using a task in which they serve as cues and not distractors. In this important way, our results show a high degree of similarity in covert and overt automated, voluntary, and combined attention when the tasks and stimuli are equated across the two response conditions. Future studies are needed to examine whether combined attention would also emerge in overt orienting when automated orienting is elicited with arrows that are manipulated as distractors in a voluntary saccade task.

A natural question arising from these data concerns the notion of whether the neural mechanisms that may support combined effects in covert and overt attention may also be similar. While there is a high degree of overlap between neural activity observed for covert and overt attentional conditions [11,33,34,35,59], this overlap is not complete [35]. Furthermore, even when the same brain areas have been found to be active, differential relative levels of activity have been observed depending on the type of attentional orienting [11,33,35,36]. In general, studies that have examined event related potentials (ERP), as well as steady state visual evoked potentials (SSVEP) in response to shifting and maintaining attention, tend to show an overall greater degree of neural activity, for example, in terms of activations in the precentral sulcus, intraparietal sulcus, and lateral occipital cortex and modulation of SSVEP amplitudes associated with overt, relative to covert attention [33,36]. Patient studies show that the frontal eye fields (FEF) appear to be one of the most likely areas to have differential effects on overt and covert orienting, as lesions of this part of the brain have been found to disrupt voluntary but not reflexive overt orienting, while, on the other hand, showing a disruption of reflexive covert attention [60].

Using EEG, we have recently shown that combined covert attention was most strongly associated with changes in power at the alpha frequency [27], such that the greatest alpha synchrony was observed in the combined condition in response to the appearance of invalidly cued targets, which is typically indicative of attentional suppression. One might predict that similar changes in alpha power may be even greater for overt attention. This is because one of the predominant differences in neural activity for overt relative to covert orienting is a larger overall change in activity associated with overt orienting in terms of overall EEG activity, SSVEP amplitudes, as well as BOLD responses in fMRI within overlapping areas of the overt and covert attentional networks [11,33,35,36]. While increased frontal and pre-frontal EEG activation during covert relative to overt orienting may reflect saccade inhibition [59], greater BOLD activation in occipital areas during overt orienting [35] may reflect greater attentional inhibition. As increased alpha activity has been correlated with increased attentional inhibition in general (e.g., References [61,62]), and more recently with the operation of combined attention [27], this increased occipital activity may reflect an even greater degree of alpha activity increase and additional attentional suppression when attention is directed in an overt relative to covert fashion. Specifically, attention may undergo stricter gating such that it is suppressed for irrelevant locations to facilitate focus at potential response-relevant target locations [63]. Future studies in which eye tracking and EEG and/or fMRI measures are integrated are needed to assess these outstanding questions.

In sum, the present study assessed whether attention combined similarly in covert and overt conditions. Consistent with past research demonstrating similarities in behavioral and neural data for overt and covert orienting, the same pattern of behavioral responding in combined attention was found for covert and overt conditions. This result points both to the general flexibility of these attentional systems and suggests that combined attention in overt conditions may rely in part on the similar functional mechanisms of suppression reported for covert conditions.

## References

[1] Klein R., Kingstone A., Pontefract A., Rayner K. (1992). Orienting of Visual Attention. Eye Movements and Visual Cognition.

[2] Posner P. (1980). Orienting of attention. Q. J. Exp. Psychol..

[3] Posner P. (2016). Orienting of attention: Then and now. Q. J. Exp. Psychol..

[4] Jonides J., Long J., Baddeley A. (1981). Volntary versus automatic control over the mind’s eye. Attention and Performance IX.

[5] Müller H.J., Findlay J.M. (1988). The effect of visual attention on peripheral discrimination thresholds in single and multiple element displays. ACTA Psychol..

[6] Müller H.J., Rabbitt P.M.A. (1989). Reflexive and voluntary orienting of visual attention: Time course of activation and resistance to interruption. J. Exp. Psychol. Hum..

[7] Bobak A.K., Langton S.R.H. (2015). Working memory load disrupts gaze-cued orienting of attention. Front. Psychol..

[8] Hunt A.R., Kingstone A. (2003). Inhibition of return: Dissociating attentional and oculomotor components. J. Exp. Psychol. Hum..

[9] Kuhn G., Tatler B.W., Cole G.G. (2009). You look where I look! Effect of gaze cues on overt and covert attention in misdirection. Vis. Cogn..

[10] Groner R., Groner M.T. (1989). Attention and eye movement control: An overview. Eur. Arch. Psychiatr. Neurol. Sci..

[11] de Haan B., Morgan P.S., Rorden C. (2008). Covert orienting of attention and overt eye movements activate identical brain regions. Brain Res..

[12] Emery N.J. (2000). The eyes have it: The neuroethology, function and evolution of social gaze. Neurosci. Biobehav. Rev..

[13] Friesen C.K., Kingstone A. (1998). The eyes have it! Reflexive orienting is triggered by nonpredictive gaze. Psychon. Bull. Rev..

[14] Shepherd S.V. (2010). Following gaze: Gaze-following behavior as a window into social cognition. Front. Integr. Neurosci..

[15] Zuberbühler K. (2008). Audience effects. Curr. Biol..

[16] Sali A.W., Anderson B.A., Yantis S. (2014). The role of reward prediction in the control of attention. J. Exp. Psychol. Hum..

[17] Awh E., Belopolsky A.V., Theeuwes J. (2012). Top-down versus bottom-up attentional control: A failed theoretical dichotomy. Trends Cogn. Sci..

[18] Kadel H., Feldmann-Wüstefeld T., Schubö A. (2017). Selection history alters attentional filter settings persistently beyond top-down control. Psychophysiology.

[19] Kingstone A., Friesen C.K., Gazzaniga M.S. (2000). Reflexive joint attention depends on lateralized cortical connections. Psychol. Sci..

[20] Ristic J., Friesen C.K., Kingstone A. (2002). Are eyes special? It depends on how you look at it. Psychon. Bull. Rev..

[21] Ristic J., Landry M. (2015). Combining attention: A novel way of conceptualizing the links between attention, sensory processing, and behavior. Atten. Percept. Psychophys..

[22] Ristic J., Landry M., Kingstone A. (2012). Automated symbolic orienting: The missing link. Front. Psychol..

[23] Ristic J., Kingstone A. (2012). A new form of human spatial attention: Automated symbolic orienting. Vis. Cogn..

[24] Hayward D.A., Ristic J. (2015). Exposing the cuing task: The case of gaze and arrow cues. Atten. Percept. Psychophys..

[25] Hayward D.A., Ristic J. (2016). Automated symbolic orienting is not modulated by explicit temporal attention. Acta Psychol..

[26] Hayward D.A., Ristic J. (2018). Changes in tonic alertness but not voluntary temporal preparation modulate the attention elicited by task-relevant gaze and arrow cues. Vision.

[27] Blair C.D., Ristic J. (2018). Combined attention controls complex behavior by suppressing unlikely events. Brain Cogn..

[28] Olk B., Cameron B., Kingstone A. (2008). Enhanced orienting effects: Evidence for an interaction principle. Vis. Cogn..

[29] Olk B., Tsankova E., Petca A.R., Wilhelm A.F. (2014). Measuring effects of voluntary attention: A comparison among predictive arrow, colour, and number cues. Q. J. Exp. Psychol..

[30] Ristic J., Kingstone A. (2006). Attention to arrows: Pointing to a new direction. Q. J. Exp. Psychol..

[31] Sheliga B.M., Craighero L., Riggio L., Rizzolatti G. (1997). Effects of spatial attention on directional manual and ocular responses. Exp. Brain Res..

[32] Shepherd M., Findlay J.M., Hockey R.J. (1986). The relationship between eye movements and spatial attention. Q. J. Exp. Psychol. A.

[33] Beauchamp M.S., Petit L., Ellmore T.M., Ingeholm J., Haxby J.V. (2001). A parametric fMRI study of overt and covert shifts of visuospatial attention. Neuroimage.

[34] Corbetta M. (1998). Frontoparietal cortical networks for directing attention and the eye to visual locations: Identical, independent, or overlapping neural systems?. Proc. Natl. Acad. Sci. USA.

[35] Corbetta M., Akbudak E., Conturo T.E., Snyder A.Z., Ollinger J.M., Drury H.A., Linenweber M.R., Petersen S.E., Raichle M.E., Van Essen D.C. (1998). A common network of functional areas for attention and eye movements. Neuron.

[36] Walter S., Quigley C., Andersen S.K., Mueller M.M. (2012). Effects of overt and covert attention on the steady-state visual evoked potential. Neurosci. Lett..

[37] Faul F., Erdfelder E., Lang A.G., Buchner A. (2007). G*Power 3: A flexible statistical power analysis program for the social, behavioral and biomedical sciences. Behav. Res. Methods.

[38] Cohen F. (1988). Statistical Power Analysis for the Behavioral Sciences.

[39] Rosenthal R. (1991). Meta-Analytic Procedures for Social Research.

[40] Brainard D.H. (1997). The Psychophysics Toolbox. Spat. Vis..

[41] Pelli D.G. (1997). The VideoToolbox software for visual psychophysics: Transforming numbers into movies. Spat. Vis..

[42] Hayward D.A., Voorhies W., Morris J.L., Capozzi F., Ristic J. (2017). Staring reality in the face: A comparison of social attention across laboratory and real world measures suggests little common ground. Can. J. Exp. Psychol..

[43] Hommel B., Pratt J., Colzato L., Godijn R. (2001). Symbolic control of visual attention. Psychol. Sci..

[44] Ristic J., Kingstone A. (2009). Rethinking attentional development: Reflexive and volitional orienting in children and adults. Dev. Sci..

[45] Tipples J. (2002). Eye gaze is not unique: Automatic orienting in response to uninformative arrows. Psychon. Bull. Rev..

[46] Capozzi F., Becchio C., Willemse C., Bayliss A.P. (2016). Followers are not followed: Observed group interactions modulate subsequent social attention. J. Exp. Psychol. Gen..

[47] Dienes Z. (2011). Bayesian versus orthodox statistics: Which side are you on?. Perspect. Psychol. Sci..

[48] Dienes Z. (2014). Using Bayes to get the most out of non-significant results. Front. Psychol..

[49] JASP Team (2018). JASP.

[50] Rothkirch M., Madipakkam A.R., Rehn E., Sterzer P. (2015). Making eye contact without awareness. Cognition.

[51] Masson M.E.J. (2011). A tutorial on a practical Bayesian alternative to null-hypothesis significance testing. Behav. Res. Methods.

[52] Morey R.D., Rouder J.N. (2011). Bayes factor approaches for testing interval null hypotheses. Psychol. Methods.

[53] Rouder J.N., Morey R.D. (2012). Default Bayes factors for model selection in regression. Multivar. Behav. Res..

[54] Kuhn G., Benson V. (2007). The influence of eye-gaze and arrow pointing distractor cues on voluntary eye movements. Percept. Psychophys..

[55] Kuhn G., Benson V., Fletcher-Watson S., Kovshoff H., McCormick C.A., Kirkby J., Leekam S.R. (2010). Eye movements affirm: Automatic overt gaze and arrow cueing for typical adults and adults with autism spectrum disorder. Exp. Brain Res..

[56] Kuhn G., Kingstone A. (2009). Look away! Eyes and arrows engage oculomotor responses automatically. Atten. Percept. Psychophys..

[57] Kuhn G., Tewson L., Morpurgo L., Freebody S.F., Musil A.S., Leekam S.R. (2011). Developmental changes in the control of saccadic eye movements in response to directional eye gaze and arrows. Q. J. Exp. Psychol..

[58] Ricciardelli P., Bricolo E., Aglioti S.M., Chelazzi L. (2002). My eyes want to look where your eyes are looking: Exploring the tendency to imitate another individual’s gaze. Neuroreport.

[59] Kulke L., Atkinson J., Braddick O. (2016). Neural differences between covert and overt attention studied using eeg with simultaneous remote eye tracking. Front. Hum. Neurosci..

[60] Henik A., Rafal R., Rhodes D. (1994). Endogenously generated and visually guided saccades after lesions of the human frontal eye fields. J. Cogn. Neurosci..

[61] Händel B.F., Haarmeier T., Jensen O. (2011). Alpha oscillations correlate with the successful inhibition of unattended stimuli. J. Cogn. Neurosci..

[62] Hanslmayr S., Aslan A., Staudigl T., Klimesch W., Herrmann C.S., Bäuml K.H. (2007). Prestimulus oscillations predict visual perception performance between and within subjects. Neuroimage.

[63] Müller N.G., Bartelt O.A., Donner T.H., Villringer A., Brandt S.A. (2003). A physiological correlate of the “zoom lens” of visual attention. J. Neurosci..

